# Interleukin-10 (IL-10) Pathway: Genetic Variants and Outcomes of HIV-1 Infection in African American Adolescents

**DOI:** 10.1371/journal.pone.0013384

**Published:** 2010-10-14

**Authors:** Sadeep Shrestha, Howard W. Wiener, Brahim Aissani, Wei Song, Aditi Shendre, Craig M. Wilson, Richard A. Kaslow, Jianming Tang

**Affiliations:** 1 Department of Epidemiology, University of Alabama at Birmingham, Birmingham, Alabama, United States of America; 2 Department of Medicine, University of Alabama at Birmingham, Birmingham, Alabama, United States of America; University of Cape Town, South Africa

## Abstract

**Background:**

Immunological and clinical outcomes can vary considerably at the individual and population levels during both treated and untreated HIV-1 infection. Cytokines encoded by the interleukin-10 gene (*IL10)* family have broad immunomodulatory function in viral persistence, and several SNPs in the *IL10* promoter sequence have been reported to influence pathogenesis or acquisition of HIV-1 infection.

**Methodology/Principal Findings:**

We examined 104 informative SNPs in *IL10, IL19, IL20, IL24, IL10RA* and *IL10RB* among 250 HIV-1 seropositive and 106 high-risk seronegative African American adolescents in the REACH cohort. In subsequent evaluation of five different immunological and virological outcomes related to HIV-1 infection, 25 SNPs were associated with a single outcome and three were associated with two different outcomes. One SNP, rs2243191 in the *IL19* open reading frame (Ser to Phe substitution) was associated with CD4^+^ T-cell increase during treatment. Another SNP rs2244305 in *IL10RB* (in strong linkage disequilibrium with rs443498) was associated with an initial decrease in CD4^+^ T-cell by 23±9% and 29±9% every 3 months (for AA and AG genotypes, respectively, compared with GG) during ART-free period. These associations were reversed during treatment, as CD4^+^ T-cell increased by 31±0.9% and 17±8% every 3 months for AA and AG genotype, respectively.

**Conclusions/Significance:**

In African Americans, variants in *IL10* and related genes might influence multiple outcomes of HIV-1 infection, especially immunological response to HAART. Fine mapping coupled with analysis of gene expression and function should help reveal the immunological importance of the *IL10* gene family to HIV-1/AIDS.

## Introduction

The *IL10* gene has been implicated in HIV-1 infection and pathogenesis in numerous studies [1], [2], [3], [4]. IL-10 is a cytokine produced mainly by T_H_2 cells and occasionally by activated B-cells, T_H_1 cells, activated macrophages, and nonhematopoietic cells (e.g., keratinocytes) [5], [6]. It has a uniquely broad immunomodulatory effect, especially in suppressing cell-mediated immunity through down-regulating pro-inflammatory cytokines, co-stimulatory molecules, as well as major histocompatibility complex (MHC) class II proteins. Animal models suggest that IL-10 plays a significant role in viral persistence[7] in general and has specific effects on HIV-1 infection through T-cell activity [8], [9]. IL-10-positive CD8^+^ T cells have a regulatory role in the immune dysfunction of HIV-1 infection [10]. IL-10 levels are greater in HIV-1-positive individuals with declining CD4^+^ T-cell and high HIV-1 viral loads than in newly infected individuals[11]. In contrast, long-term non-progressors have low IL-10 levels comparable to HIV-1-negative individuals. The HIV-1 regulatory protein, Tat, has also been shown to induce IL-10 production [12]. Higher concentrations of IL-10 appear to limit HIV-1 replication *in vivo* by inhibiting macrophages/monocytes and T-cell lymphocyte replication [13], [14], [15], [16], and administration of IL-10 to HIV-1-positive patients has also been shown to decrease the number of circulating HIV-1 virions [17]. IL-10 inhibition of T-cell apoptosis could actually be beneficial for HIV-1-infected individuals [18]. IL-10 may also selectively up-regulate the expression of the CC chemokine receptors CCR5, CCR2 and CCR1 in human monocytes [19], [20], [21]; these control entry of the virus into the cell. Further, several studies indicate that IL-10 production is induced *in vivo* [22] and *in vitro* [23] during HIV-1 infection.

The association between SNPs in *IL10* and HIV-1 pathogenesis or infection has received considerable attention, but the importance of genotypic variation remains unclear. In a multi-cohort analysis [24], European Americans carrying the A allele at the *−592* position *(*rs1800872) of *IL10* promoter were at increased risk for HIV-1 infection (OR = 1.75, p = 0.03). Investigators in that same study suggested that 25–30% of untreated, long-term non-progressors (>10 years without AIDS) among European Americans could be explained by the presence of the wild-type rs1800872 C/C genotype. Additional *IL10* variants have also been associated with disease progression to AIDS [2], [24], [25], often with inconsistent conclusions.

Selective adaptation of polymorphisms within *IL10* in different populations has resulted in semi-conserved regions and generated these adjacent paralogs that may have entirely or only partially redundant or antagonistic functional properties. *IL19*, *IL20*, and *IL24* are located upstream of the *IL10* gene within the cytokine gene cluster in a 200 kb region on chromosome 1q31**–**32 (Figure S1). The four genes have similar intron–exon structures and they form a subfamily based on similar protein sequences, cellular sources (i.e., activated immune cells, including monocytes), and receptors on target cells. Several functional and biological interactions are known between these paralogs. For example, IL-19 stimulation can increase its own expression *and IL10* mRNA transcription, while IL-10 potently down-regulates the ‘autoinduction’ of IL-19 [26], [27]. Upregulation of IL-19 and IL-20 has been found following *in vitro* stimulation of mammary and amniotic epithelial cells with extracellular HIV-1 Tat [28]. Similarly, IL-10 utilizes the *JAK-STAT* pathway for downstream signaling, and the paralogs IL-19 *and* IL-20 have capacity to induce phosphorylation of STAT1 or STAT3 or both [29]. Overall, immune regulation mediated by the *IL10* gene family can influence HIV-1 pathogenesis through multiple mechanisms. Genotypes of *IL10* and the evolutionarily conserved regulatory regions encoded by the related gene sequences could become useful biomarkers if their relationships to HIV-1 infection and disease progression with our without treatment are unambiguously established. Here, we hypothesize that involvement of the IL-10 molecule in HIV-1 infection and pathogenesis could be modulated through complex interactions among *IL10* and its related genes, especially those (*IL19*, *IL20* and *IL24*) in the flanking genomic region and the IL-10 receptor genes (*IL10RA* and *IL10RB*).

## Materials and Methods

### Ethics Statement

The parent study and this sub-study conformed to the procedures for informed written consent (parental permission was obtained wherever required) approved by institutional review boards (IRB) at all sponsoring organizations and to human-experimentation guidelines set forth by the United States Department of Health and Human Services and finally reviewed and approved by the UAB IRB.

### Study population

We studied 356 eligible African American participants from the Reaching for Excellence in Adolescent Care and Health (REACH) study. The characteristics of the cohort, recruitment, follow-up and research objectives are described in detail elsewhere [30], [31]. Briefly, REACH is an observational study of 352 HIV-1 seropositive (based on self-report vertical transmissions were not included) and comparable 196 high-risk seronegative adolescents (13 to 19 years old) recruited from 15 clinical sites in 13 US cities between 1996 and 1998 and followed until 2001. The cohort comprised 71% African Americans and 76% females. All subjects designated uninfected for this study had a history of either sexual intercourse or injection drug use, had a negative HIV-1 enzyme linked immunosorbent assay (HIV-1-ELISA) performed within 30 days of enrollment into REACH, and remained seronegative until the end of the study period. All infected subjects had a positive ELISA, with a confirmatory Western blot performed before REACH enrollment. Participants were followed on a quarterly basis for epidemiologic, clinical, and laboratory evaluations, including documentation of demographic and risk behaviors, collection of medical history and various biological samples, along with tests for HIV-1 infection, other sexually transmitted infections, CD4^+^ T-cell, and immunological outcomes. HIV-1 RNA concentration (viral load, VL) and immunological outcomes were measured every three months. CD4^+^ T-lymphocytes were quantified by flow cytometry in National Institute of Allergy and Infectious Disease (NIAID)-certified laboratories at each clinical site. Viral load (VL) was measured in a centralized laboratory using either nucleic acid sequence-based amplification (NASBA, lower limit detection  = 400 copies/mL) or NucliSens assays (Organon Teknika, Durham, NC, lower limit  = 80 copies/mL). Only two individuals seroconverted during follow-up of REACH and all others were HIV-1 seropositive at baseline (enrollment). In most cases, duration of infection, as inferred from young age (median at 17), self-reported sexual debut (not earlier than age 13), and other risk behaviors (no vertical transmission), should be no more than five years when viral load measures were taken. As a result, stable VL at two or more clinical visits was treated as a proxy for VL set-point [30], [32].

### Study design

To minimize confounding by population stratification, our analyses were restricted to the subset of African Americans. Small numbers representing additional ethnic groups (Hispanics, European Americans, and others or unknown) were not tested because of limited statistical power.

### HIV-1 infection

We analyzed 250 HIV-1 seropositive (199 females and 51 males) and 106 high-risk seronegative (84 females and 22 males) African American adolescents.

### Viral load set-point

As we previously described [33], viral load set-point was estimated using the covariance structure model of the first 2–4 sequential viral load measures across individual participants. We included each individual as a random effect variable and excluded the visits where the subsequent viral loads did not appear to be in steady state, i.e. inter-visit viral load variation exceeded 0.5 log_10_ copies per ml of plasma. Of the 250 HIV-1-positive African American subsets, 121 (102 females and 19 males) had VL set-point.

### Categorical Disease Progression

Three ordinal patient groups were classified according to longitudinal immunological and virological data: 26 participants were defined as “controllers” (VL<1000 copies/ml and CD4^+^ T-cell >450×10^6^ cell/l), 36 as “non-controllers” (VL>16,000 copies/ml, CD4^+^ T-cell <450×10^6^ cell/l), and 99 as “intermediates” [34].

### CD4^+^ T-cell trajectories

The availability of longitudinal CD4^+^ T-cell provided the opportunity to determine the influence of genetic variants on CD4^+^ T-cell slope during antiretroviral therapy (ART)-free and HAART-using periods. We first examined the CD4^+^ T-cell trajectory among 148 individuals during ART-free visits (median visit  = 4 [IQR 3**–**8]). Next, we assessed the CD4^+^ T-cell trajectory among169 adolescents during the period that they received HAART with self-reported adherence (median visit  = 5 [IQR 3**–**9]) [30]. All AIDS-free individuals who had follow-up data during at least 2 consecutive visits were included in the analyses. Consistent with the Public Health Service Guidelines for the Use of Antiretroviral Agents in HIV-Infected Adults and Adolescents [35], participants who were prescribed a combination of two nucleoside reverse transcriptase inhibitors and either a protease inhibitor, or a non-nucleoside reverse transcriptase inhibitor, or a zidovudine/lamivudine combination regimen plus another antiretroviral drug, at the time of a study visit were considered eligible for analysis. Data on HAART were obtained through interviews and chart reviews for past and current prescriptions, and adherence data were obtained through interviews, as described elsewhere [36]. All participants were censored at the last available visit (including end of study) or the first visit that they reported not adhering to HAART regimen. Individuals who did not receive HAART were excluded even if they received suboptimal treatment. Of note, a subset of ART-naive individuals with adequate follow-up started HAART during the later phase of study and they were also eligible for analyses of HAART-induced changes in CD4^+^ T-cell slope.

### Genotyping

High-molecular-weight genomic DNA extracted from whole blood was used for the genotyping of 115 SNPs throughout the coding and UTR regions, including haplotype tagging-SNPs in introns of *IL10* and related genes for three paralogs (*IL19*, *IL20* and *IL24*), and two receptor (*IL10RA* and *IL10RB*). The custom GoldenGate assay (Illumina, San Diego, CA) and iPLEX assay (Sequenom massarray) were done using two commercial platforms (Illumina, San Diego, CA and Broad Institute, Cambridge, MA).

### Analyses of SNP genotypes

Hardy-Weinberg equilibrium (HWE) was examined for each SNP. Pairwise linkage disequilibrium (LD) was quantified using the absolute value of Lewontin's D' and r^2^ and LD plots were generated using Haploview version 4.1 [37]. Separate analyses were conducted for each outcome of the HIV-1 pathogenesis stages and only previously confirmed and validated (in other cohorts) genetic and non-genetic factors were adjusted for in the analyses.

Chi-square tests were used to assess the distribution of alleles and genotypes between HIV-1-positive and HIV-1-negative high-risk adolescents and all significant associations (p<0.05) were further evaluated accounting for non-genetic factors age, gender and number of sex partners as previously described [38]. For analysis of VL set-point, we estimated mean VL [39] by genotype at each SNP, and also accounted for the confirmed HLA-B57 effect [33] and multiple comparisons using Tukey-Kramer adjustment. We also determined the allelic and genotypic heterogeneity among controllers, non-controllers, and intermediates controllers, as defined previously [34]. Categorical outcomes were assessed by *χ^2^* and Cochran-Armitage trend tests and analyses were also performed separately for HLA-B57 negative individuals since this was such a strong marker in previous analyses.

Changes in CD4^+^ T-cell over time were assessed using the linear mixed random effects models assuming a Poisson regression for CD4^+^ T-cell where the parameter *λ* is the expected value or mean of the distribution. From the definition of the distribution, this parameter must have a positive value, so a regression model was set up in log function as follows: *log(λ)  =  β_0_ + β_1_x_1_+ β_2_x_2_* for the case of two predictor variables where *λ*  =  exp (*β0 + β_1_x_1_+ β_2_x_2_*) and subsequently, **B**_i_  =  exp (*βi)* for i  = 0, 1, 2 and the count predicted by the model is given by 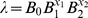. Therefore, an increase in *x_1_* of one unit causes the expected count to be multiplied by **B**_1_  =  exp (β_1_), and similarly an increase of one unit of *x_2_* causes the expected count to be multiplied by **B**_2_  =  exp (β_2_). These models accounted for the correlation between serial measurements at consecutive visits from each person and were implemented using the Proc routine in SAS. To account for any variability in the trajectory based on the initial CD4^+^ T-cell, the model included an adjustment for baseline CD4^+^ T-cell. Additionally, to account for the potential confounding effects of HAART, we fit the random effects model separately for both treatment-free intervals and treatment time periods. All statistical analyses were limited to univariate model and were performed using SAS version 9.2 (SAS Institute, Cary, NC).

## Results

Of the 115 SNPs with genotyping results, six did not meet HWE and 5 had minor allele frequency less than 2%, leaving 104 informative SNPS (15 in *IL10*, 23 in *IL19*, 13 in *IL20*, 9 in *IL24*, 18 in *IL10RA* and 26 in *IL10RB*). Patterns of LD (r^2^) are shown for these SNPs in *IL10* and neighboring paralogs at chromosome 1q31–32 (Supplementary Figure S1a), *IL10RA* at 11q23 (Supplementary Figure S1b) and *IL10RB* at 21q22 (Supplementary Figure S1c).

### HIV-1 Infection

One SNP in *IL10RB (*rs2266590 in intron; 4 p = 0.05) and one in *IL20 (*rs2981572 in 5′UTR; p = 0.006*)* were associated with risk of HIV-1 infection (Table 1). In particular, a protective effect was associated with minor allele of rs2981572 (OR = 0.5 [CI: 0.21–0.94], p = 0.006). Even after adjusting for the non-genetic factors, age, sex and number of sex partners, the effect of this SNP was strongly protective.

**Table 1:** Frequency distribution of genotypes in *IL10* related genes between HIV-1^+^ and high risk HIV-1^−^ adolescents.

Gene and SNP (rs#)	Frequencies	Genotype	Allelic p-value	Trend p-value
	HIV-1^+^ vs HIV-1^−^ N = (250) (106)	p adjusted-p*		
***IL10RB***				
** rs2266590**			0.05	0.01
** AA**	0.10 0.06	0.03 0.07		
** AG**	0.45 0.36	0.04 0.06		
** GG**	0.45 0.59	-		
***IL20***				
** rs2981572**			**0.006**	**0.003**
** TT**	**0.15 0.30**	**0.0009 0.006**		
** TG**	**0.52 0.45**	**0.03 0.05**		
** GG**	**0.33 0.25**	**- -**		

* adjusted for gender, age at baseline and life-time sex partner.

### HIV-1 Viral Load Set-point

Three SNPs in *IL10* (rs1800890, rs2222202 and rs1878672, with the latter two in LD) were associated with significant differences in mean VL in the univariate model by genotype (Table 2, p<0.005–0.04). Specifically, the mean VLs were –log 3.74 (±0.09), 3.51 (±0.11), and 2.82 (±0.36) cp/ml for TT, AT, and AA genotypes respectively for rs1800890. For the two SNPs in LD (represented by rs2222202 based on the more complete genotype data), the mean VLs were –log_10_ 3.76 (±0.10), 3.55 (±0.10), and 2.85 (±0.26) cp/ml for GG, AG, and AA genotypes respectively (p = 0.03 for trend). After adjusting for the HLA B*57 effect as previously reported [33], rs2222202 (rs1878672) still had significant effect (p = 0.02) where the mean VLs were –log_10_ 3.40 (±0.13), 3.24 (±0.10), and 2.65 (±0.26) cp/ml for GG, AG, and AA genotypes, respectively.

**Table 2:** Difference in viral-load set-point by genotype in *IL10* gene family.

Gene and SNP (rs#)	Genotype (*n*)	-log_10_ VL (cp/ml) mean(±std)	p-value	adjusted* mean(±std)	adjusted* p-value
***IL10***					
** rs1800890**	AA (5)	2.42 (±0.36)	0.001		
	AT (46)	3.51 (±0.11)	0.01		ns
	TT (70)	3.74 (±0.09)	-		
** rs2222202**	AA (9)	**2.93 (±0.25)**	**0.008**	**2.65 (±0.25)**	**0.02**
** (rs1878672)**	AG (50)	**3.55 (±0.10)**	**0.03**	**3.24 (±0.12)**	**0.08**
	GG (62)	**3.74 (±0.10)**	-	**3.40 (±0.13)**	-

rs2222202 and rs1878672 are in LD (*r* ^2^ = 1) and the data are presented for rs222202 with more complete genotype data; ns =  not statistically significant.

* adjusted for HLA-B57.

### Categorical Disease Progression

**Figure 1:**
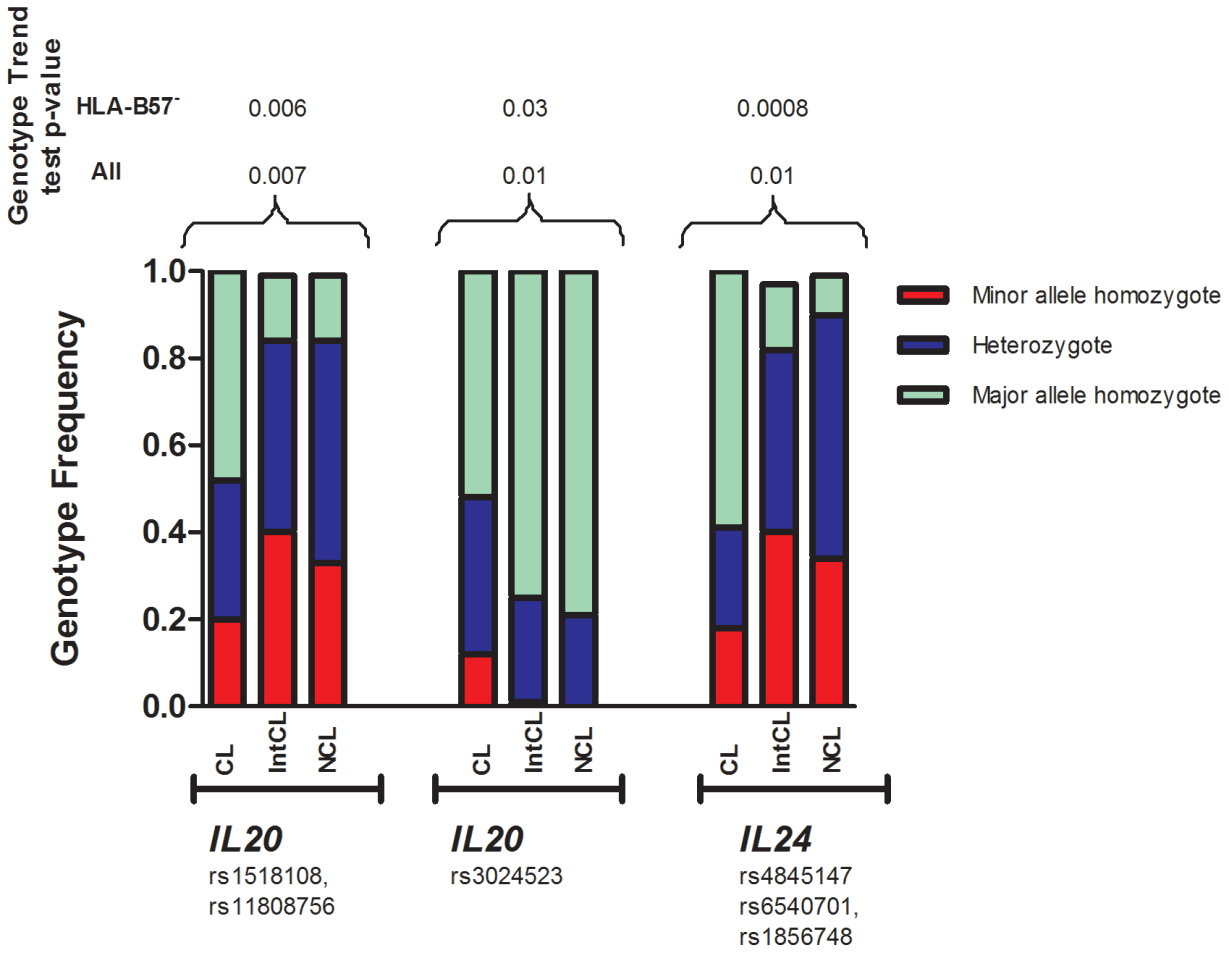
SNPs in *IL10* gene family associated with the combined immunological and virological control of HIV infection. Genotype frequencies are compared for all “controllers” (CLs), “intermediate controllers” (IntCL) and “non-controllers” (NCL) as defined by virological and immunological markers. Two SNPs in *IL20* (rs1518108 and rs11808752) are in LD (*r* ^2^ = 0.90) and data is presented for rs1518108 with more complete data; rs4845147, rs6540701 and rs1856748 in *IL24* are in LD (*r* ^2^ = 0.91) and data are presented for rs4845147 with more complete data; p-value is presented for all individuals including and excluding 12 HLA-B57+ individuals.

As shown in Figure 1, associations (*p* = 0.0008**–**0.03) were observed for genotype trend test among the three patient groups with two SNPs in the 3′ UTR and one SNP in the 5′ UTR of *IL20 (*rs1518108, rs11808756, rs3024523, where the first 2 were in LD, *r* ^2^>0.90) and b) three SNPs in the 5′ UTR of *IL24 (*rs4845147, rs6540701, rs1856748, where all were in complete LD). Interestingly, it seems that the controllers had a higher frequency (0.12) of CC genotype for rs3024523, which is rare or absent in the other two groups. When all 12 individuals with HLA-B57 (all are “controllers”) were excluded from analysis, there were still statistically significant trends for differential SNP genotype distributions across the three ranked patient categories (“controllers” to “intermediates” to “non-controllers”).

### Rates of CD4^+^ T-cell change

Among the AIDS-free untreated adolescents, the CD4^+^ T-cell trajectory from one visit to the next in the absence of any ART therapy (i.e. every 3 months on average) was associated with multiple SNP genotypes. Five SNPs in *IL19* (rs908704, rs4845143, rs2243174, rs2243188, rs2243193, where the first 2 and the latter 3 were in LD, *r* ^2^>0.90), one in *IL24* (rs291111), one in *IL10RA* (rs2244305), and 2 in *IL10RB* (rs2244305, rs2243498) showed significant differences (Table 3 top panel). Of note, rs29111 had the greatest changes where the CD4^+^ T-cell decreased by 62±18% and 66±19% per 3 months for genotypes TT and TC, respectively, compared to CC.

**Table 3:** Influence of *IL10* gene family genotypes on CD4^+^ T-cell trajectory during a) ART-free period (n = 148) and b) HAART (n = 169).

Gene and SNP (rs#)	Genotype (*n*)	CD4^+^ decline ±95% CI (% decrease every 3 months)	p-value
**ART-Free** ***IL19***			
** rs908704**	AA (17)	−31±8	0.0002
** (rs4845143)**	AG (60)	−19±8	0.02
** rs2243174**	AA (25)	−31±7	2.9*10e^−5^
** (rs2243188)**	AG (69)	−17±7	0.02
** (rs2243193)**			
***IL24***			
** rs291111**	TT (8)	−62±19	0.001
	TC (56)	−66±9	0.0008
***IL10RA***			
** rs9610**	AA (18)	−29±9	0.002
	AG (58)	−24±9	0.009
***IL10RB***			
** rs2244305**	AA (23)	−23±9	0.008
** (rs2243498)**	AG (69)	−29±9	0.001
**On HAART** ***IL10***			
** rs3024496**	TT (18)	23±10	0.03
	TC (73)	31±9	0.002
***IL19***			
** rs2243191**	TT (7)	21±10	0.04
	TC (53)	19±6	0.004
***IL24***			
** rs291107**	AA (7)	56±12	1.9*10e^−6^
	AG (50)	20±6	0.0004
** rs291109**	AA (12)	20±8	0.008
	AG (46)	27±6	2.3*10e^−5^
***IL10RB***			
** rs2244305**	AA (23)	31±9	0.0003
** (rs2243498)**	AG (69)	17±8	0.02

rs908704 and rs4845143 are in LD (*r* ^2^ = 0.91) and data are presented for rs908704, which has more complete data; rs2243174, rs2243188 and rs2243193 are in LD (*r* ^2^>0.90) and data are presented for rs2243174, which has more complete data; rs2244305 and rs2243498 are in LD (r^2^>0.94) and data are presented for rs2244305, which has more complete data.

In the 169 individuals with multiple treated visits, the CD4^+^ T-cell trajectory in response to therapy deviated significantly by multiple SNP genotypes (Table 3 bottom panel). In particular, one SNP in *IL10* (rs3024496), one in *IL19* (rs2243191), two in *IL24* (rs291107, rs291109), and two in *IL10RB* (rs2244305, rs2243498 in LD) showed statistically significant differences in the CD4^+^ T-cell trajectory over time with HAART. A known functional SNP, rs2243191 (encoding a Phe to Ser substitution) in exon 1 of *IL19* showed a change where the CD4^+^ T-cell increased by 21±10% and 19±6% cells every 3 months for genotypes TT and TC, respectively, compared to CC, suggesting a dominant effect. Overall, polymorphisms in six related genes could be associated with five HIV-1-related outcomes in our study population (Table 4).

**Table 4:** Summary of genetic influence of IL10 gene family and various HIV infection outcomes.

Gene andSNP (rs#)	Polymorphisms	Location	MAF	HIV infection	Viral load set-point	Viral and immune controllers	ART-free CD4+ T-cell slope	During-HAARTCD4+ T-cell slope
Sample size			356 AA	250 HIV+106 HIV^−^	121	26 controllers36 non-controllers99 intermediates	148	169
***IL10*** ** rs1800890** ** rs2222202^ a+^** ** rs1878672^ a◊^** ** rs3024496** +	A/TA/GC/GC/T	5′UTRintronintron3′UTR	0.220.280.280.39		✓✓✓✓✓✓			✓✓
***IL19*** ** rs4845143^b+◊^** ** rs2243174^c◊^** ** rs908704^b^** ** rs2243188^ c+^** ** rs2243193^c+^** ** rs2243191** +	C/TG/AG/AC/AG/AT/C	intronintronintronintron3′UTRNon-syn(Ser>Phe)	0.320.400.330.400.410.17				✓✓✓✓✓✓✓✓✓✓	✓✓
***IL20*** ** rs1518108^d^** ** rs11808756** * ** rs3024523** ** rs2981572^d^**	C/TT/CG/AG/T	3′UTR5′UTR5′UTR5′UTR	0.440.170.150.44	✓✓		✓✓✓		
***IL24*** ** rs6540701^e^** ** rs1856748^e+^** ** rs291109** ** rs291107** + ** rs291111** ◊ ** rs4845147**	T/CA/GG/AG/AC/TC/T	5′UTR5′UTRintronintron5′UTR5′UTR	0.360.360.260.190.270.45			✓✓✓✓✓✓	✓✓	✓✓✓✓
***IL10RA*** ** rs9610**	G/A	3′UTR	0.33				✓	
***IL10RB*** ** rs8178571** * **^+^** ** rs2244305^f◊^** ** rs2266590** ◊ ** rs2243498^f^**	G/AA/GG/AG/A	3′UTRintronintronintron	0.180.370.300.36	✓		✓	✓✓✓✓	✓✓✓✓✓✓✓

Alphabetical superscripts (^a, b, c, d, e^) indicates SNPS that are in LD (*r* ^2^>0.90);

MAF = 0 in EA;

+ Illumina 1M chip;

◊ Affymetrix 6.0 chip; p-value scale: ✓ =  <0.05−0.01, ✓✓  =  <0.01−0.001, ✓✓✓  = 0.001.

## Discussion

Our study suggests that variants within the *IL10* gene pathway might influence various immunological and virological outcomes of HIV-1 infection and immunological response to HAART in African Americans. Most genetic associations with immunological and virological outcomes of HIV-1 infection have been studied in populations of European descent; relatively fewer have been evaluated in minority populations, especially African Americans, where the HIV-1/AIDS epidemic is disproportional. We observed multiple variants in the potential pathway of the *IL10* gene family, contributing individually or jointly to the heterogeneity in HIV-1 pathogenesis. These potential joint contributions demonstrate the need for pathway-oriented (systematic) approaches to immunogenetic studies either in conjunction with or even instead of analyses of single candidate genes or hypothesis free genome-wide association studies. We have examined various HIV-1/AIDS outcomes and reported several SNPs or combinations that might play complex immunological roles at various stages of HIV-1 pathogenesis.

Notably, one nonsynonymous SNP (rs2243191) that causes Ser to Phe substitution in the *IL19* open reading frame was associated with CD4^+^ T-cell trajectory during HAART. While the function of this amino acid change is still unclear, differential allelic expression combined with its function in T_H_2 pathway [40] may provide some basis for the epidemiologic findings here. Other SNPs of interest have no immediate evidence for functional properties, but their importance to gene expression (translation and mRNA stability) cannot be ruled out. Sequence alignments of human *IL10* gene and its paralogs indicate numerous regions of close homology with other species. Such conserved non-coding sequences are known to contain regulatory elements. Bioinformatics coupled with experimental approaches may eventually reveal the intrinsic connection between non-coding SNPs and gene function.

Associations of these different SNPs in different members of a “gene family” with different phenotypes of HIV-1 infection raise concern about whether the effects are of true biologic significance or are more likely to have been spuriously introduced by multiple comparisons. To avoid the latter pitfall, rather than perform multivariable, haplotype or interaction analyses that might well be underpowered in our relatively small cohort, we have only reported univariate analyses and only with adjustment for confirmed non-genetic and genetic factors, thereby presenting only a broad overview of possible associations of the *IL10* gene family with HIV-1 pathogenesis. Overall, associations with at least three SNPs could withstand Bonferroni correction for multiple (104×5 = 520) tests (corrected cut-off *p*≤9.6×10^−5^). Some of the marginal associations (in terms of borderline statistical significance levels based on relatively small sample sizes) may have been detected by chance. However, they may still provide valuable benchmarks to benefit future research, especially since the six genes have not been fully studied in any population with HIV-1 infection.

The results even from a small sample-size could possibly suggest several avenues of future inquiry. First, different genes within the *IL10* family are expected to function in different stages of HIV-1 pathogenesis. The preservation of *IL10* gene paralogs and other functionally related genes is likely the result of co-evolution, through which they act optimally in concert to confer biological effects, specifically in the context of infections with agents like HIV-1 where the antigenic repertoire evolves rapidly within the host. Second, the role of these genes may change as HIV-1 infection reaches different stages (acquisition, acute and early controlled, chronic and late progression). Selective analysis of known “tagging” SNPs in the context of one phenotype (e.g., VL) at a time is less likely to reveal more comprehensive and complex relationships. Third, involvement of the IL-10 product in immunity to HIV-1 infection could be modulated through more complex interactions among related genes that may not be physically close but occur within the same biological system. For example *IL22, IL26, IL28* and *IL29* can also use the *IL10RB* receptor [41]. Fourth, some of the SNPs we found associated with HIV-1 outcomes are polymorphic only in African-Americans. For example, three informative SNPs (Table 4) in our study population have been reported as non-polymorphic in the European American population, implicating the need to study different populations. Fifth, in the immediate post human genome project era, we have heavily relied on genome-wide association studies (GWAS) and the reported SNPs in the commercial genotyping arrays. However, of the 25 SNPs that were found to be significantly associated with different outcomes in our study (Table 4), only nine are present in the Human 1M-Duo chip (Illumina) and only 6 are present in the Affy 6.0 chip (Affymetrix), and only one is present in both panels. In other words, even the most current GWAS arrays could not sufficiently survey potentially important SNPs even if they are used in combination. More recently, GWAS has revealed that one *IL10* SNP (rs4072227) out of 14 examined was associated with HIV-1 VL set-point (adj p = 0.03) in the European American component [42], but no associations surfaced in the African Americans. Further, rs4072227 does not seem to be in LD with any functional SNP in the region in any of the HapMap populations. Finally, we have noted that other immune response genes reside in the neighborhood of *IL10* and related candidate genes selected for our work. In particular, *MAPKAP2* is only 35 kb away from the promoter of *IL10,* and some of the candidate SNPs in the former gene (mostly in noncoding sequences) may well tag other variants important to microRNA function, DNA methylation and/or gene expression.

As more detailed information about the genome becomes available, many of the poorly understood regulatory elements (SNPs in introns, 5′ and 3′UTRs) may be shown to exert more significant functional effects. Thus, reports from association studies like the present one on the *IL10* gene family as well as other genes and their related families should be weighed in the context of what is known about the biologic pathways that influence the pathogenesis of HIV-1 infection. Both population and experimental studies will be needed to evaluate the relevance of polymorphism to gene expression and immune response.

## Supporting Information

Figure S1 Pairwise linkage disequilibrium (*r* ^2^) measures for informative SNPs in a) *IL10, IL19, IL20* and *IL24* (chromosome 1q31-32), b) *IL10RA* (chromosome 11q32), and c) *IL10RB* (chromosome 21q22.11).
(0.58 MB TIF)
