# Supplementary figures and images for: Interleukin-10 (IL-10) Pathway: Genetic Variants and Outcomes of HIV-1 Infection in African American Adolescents
Source: PLoS One. 2010 Oct 14;5(10):e13384. doi: 10.1371/journal.pone.0013384 (PMC2954785; doi:10.1371/journal.pone.0013384)

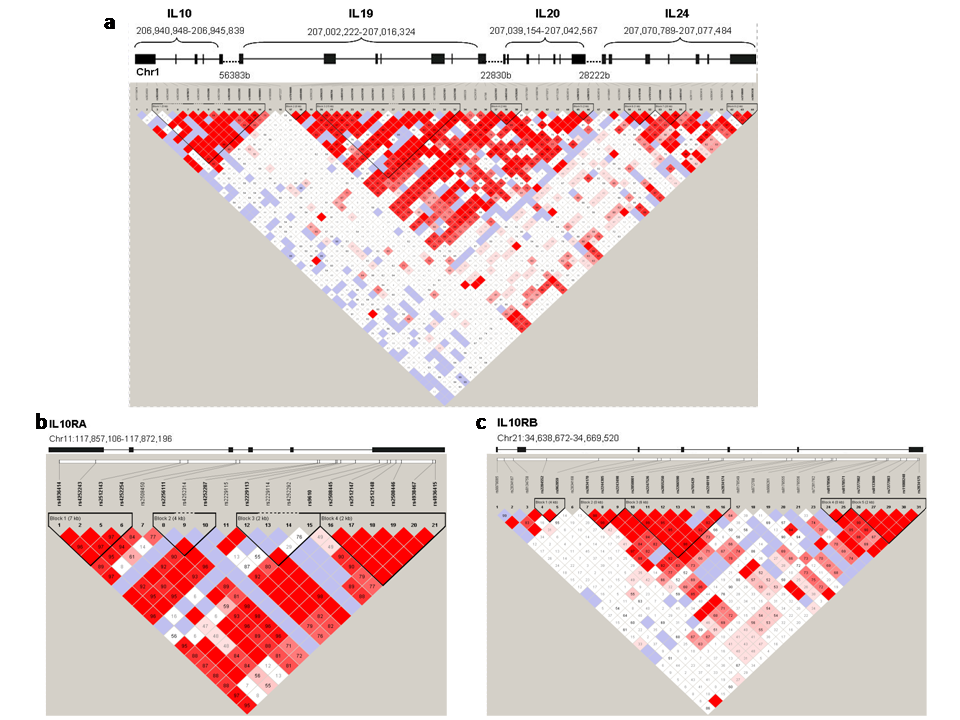

Supplement: Figure S1 — Pairwise linkage disequilibrium (r 2) measures for informative SNPs in a) IL10, IL19, IL20 and IL24 (chromosome 1q31-32), b) IL10RA (chromosome 11q32), and c) IL10RB (chromosome 21q22.11). (0.58 MB TIF) [file pone.0013384.s001.tif]
